# *Clonorchis sinensis* legumain promotes migration and invasion of cholangiocarcinoma cells via regulating tumor-related molecules

**DOI:** 10.1186/s13071-023-05694-4

**Published:** 2023-02-16

**Authors:** Yanfei Chu, Doufei Shi, Nan Wang, Lebin Ren, Naiguo Liu, Fengai Hu, Wei Meng, Sung-Jong Hong, Xuelian Bai

**Affiliations:** 1 Clinical Medicine Laboratory, Binzhou Medical University Hospital, Binzhou, 256603 Shandong People’s Republic of China; 2 Department of Geriatric Medicine, Binzhou Medical University Hospital, Binzhou, 256603 Shandong People’s Republic of China; 3 Department of Medical Environmental Biology, Chung-Ang University College of Medicine, Dongjak-Gu, Seoul, 156-756 Republic of Korea

**Keywords:** *Clonorchis sinensis*, Cslegumain, Cholangiocarcinoma, Migration and invasion

## Abstract

**Background:**

*Clonorchis sinensis* infection causes serious pathological changes in the bile duct and is highly correlated with cholangiocarcinoma. The excretory–secretory products (ESP) of *C. sinensis* play a critical role in the oncogenesis and progression of cholangiocarcinoma, while the components and precise mechanism remain unclear. Here, we evaluated the function of *C. sinensis* legumain (Cslegumain) in promoting the invasion and migration of cholangiocarcinoma cells and the mechanism involved.

**Methods:**

The structural and molecular characteristics of Cslegumain were predicted and analyzed using the online program Phyre2. Quantitative real-time polymerase chain reaction (qRT-PCR) and immunohistochemical staining were performed to test the transcriptional level of Cslegumain and its localization in the adult. Native Cslegumain was detected by western blotting assay. The effects of Cslegumain on the proliferation, invasion and migration of cholangiocarcinoma cells were checked using CCK-8 assay, Matrigel transwell assay and scratch wound healing assay. Expression levels of tumor-related molecules regulated by Cslegumain were evaluated by qRT-PCR and western blotting assay.

**Results:**

Cslegumain showed high similarity with human legumain in the secondary and tertiary structures and displayed higher transcriptional levels in the adult worm than in the metacercariae. Native Cslegumain was detected in a catalytic form and was localized mainly in the intestine of the *C. sinensis* adult and epithelial cells of the intrahepatic bile duct. After transfection into RBE cells, Cslegumain showed high ability in promoting the invasion and migration but not the proliferation of cholangiocarcinoma RBE cells. Furthermore, the expression levels of some molecules including E-cadherin and N-cadherin were downregulated, while the levels of α-actinin 4, β-catenin and inducible nitric oxide synthase (iNOS) were upregulated.

**Conclusions:**

Our findings indicated that Cslegumain showed very similar structures as those of human legumain and could promote the invasion and migration of cholangiocarcinoma cells by regulating some tumor-related molecules.

**Graphical Abstract:**

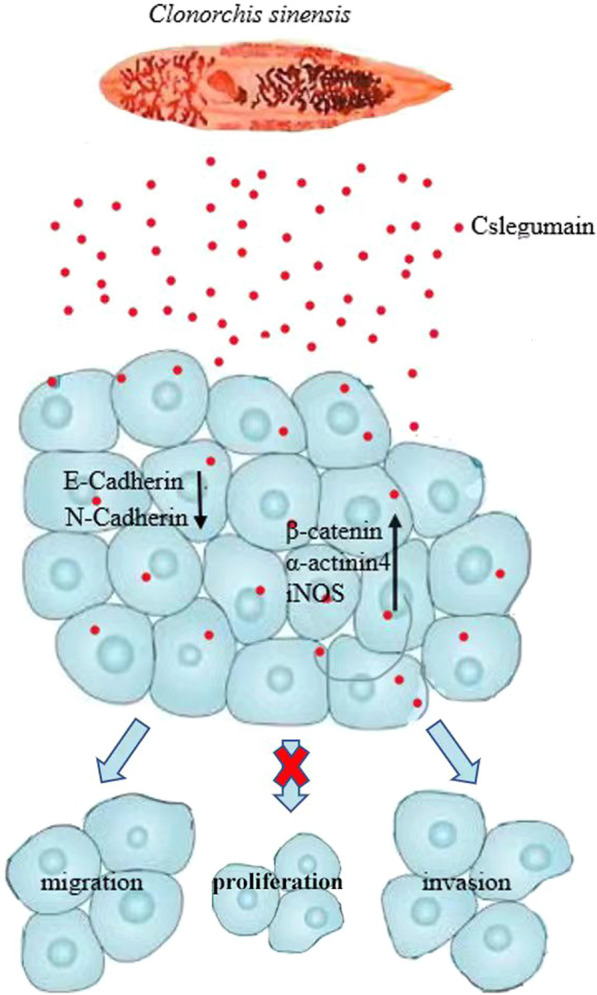

**Supplementary Information:**

The online version contains supplementary material available at 10.1186/s13071-023-05694-4.

## Background

The liver fluke, *Clonorchis sinensis*, is an important food-borne parasite endemic mainly in East Asian countries including China, Korea, Thailand and Vietnam. It presents a public health problem, as about 15 million people have been reported to be infected worldwide and 60 million are at risk of infection [1, 2]. As the final host of *C. sinensis*, mammals become infected by consuming raw or undercooked freshwater fish containing metacercariae. After being swallowed, the metacercariae excyst in the duodenum under the action of digestive juice, and the juveniles migrate into the intrahepatic bile ducts within several minutes where they develop into adults [1]. Living in the bile duct, *C. sinensis* causes serious pathological changes such as hyperplasia and inflammation of the biliary mucosa, periductal fibrosis, ductal wall thickening and even cholangiocarcinoma of the biliary tree through mechanical stimulation and excretory–secretory products (ESP) [3]. Because of its close association with cholangiocarcinoma, *C. sinensis* was classified as a biological carcinogen by the International Agency for Research on Cancer [4]. The functions and mechanisms of *C. sinensis* ESP components in the oncogenesis and development of cholangiocarcinoma have become a focus in this area. So far, the functions of various ESP components in cholangiocarcinoma have been investigated. *Clonorchis sinensis* granulin promoted the metastasis of cholangiocarcinoma and hepatocellular carcinoma by upregulating the levels of vimentin, β-catenin, matrix metalloproteinase (MMP)-2 and MMP-9 [5]. However, additional *C. sinensis* ESP components and their function mechanisms in cholangiocarcinoma still remain unclear and deserve further study.

Legumain (also called asparaginyl endopeptidase [AEP]) is a new member of the cysteine protease C13 family, with conserved structures and specifically hydrolyzing peptides on the carboxyl side of asparagine residues. The protease was originally isolated from cowpea seeds and concanavalin by Kembhavi in 1993 and subsequently was found to exist in parasites such as *Schistosoma mansoni* and *Opisthorchis viverrini* and in mammals [6]. In recent years, legumain has been found to be overexpressed in a variety of solid tumor tissues, including colorectal, breast, ovarian, prostate and gastric cancer, and was highly correlated with tumor growth, invasion and metastasis [7–11]. Thus, it was considered to be a tumor prognostic factor and a target for treatment. Legumain of *C. sinensis* (we call it Cslegumain here) was found in the ESP and could be a serodiagnostic antigen [12]. As a member of *C. sinensis* ESP, Cslegumain has high similarity with human legumain (Hlegumain), and whether it also plays an important role in cholangiocarcinoma deserves further investigation. In the present study, we describe the molecular characteristics of Cslegumain and its functions and mechanisms in promoting the migration and invasion of cholangiocarcinoma cells.

## Methods

### Ethics statement

Female 7-week-old BALB/c mice and male New Zealand White rabbits were purchased from Samtako Bio Korea Inc. (Osan, Seoul, Korea) and kept under pathogen-free conditions according to Binzhou Medical University and the Chung-Ang University animal facility. Approval for animal experiments was obtained from the Institutional Animal Care and Use Committee of Binzhou Medical University Hospital (approval number 20190104-14) and Chung-Ang University animal facility (approval number CAU-2011-0052). This study was carried out in strict accordance with the recommendations in the Guide for the Care and Use of Laboratory Animals of the Ministry of Science and Technology of the People’s Republic of China and Republic of Korea.

### Metacercariae collection, rabbit infection and recovery of* C. sinensis* adult worms

*Pseudorasbora parva* containing *C. sinensis* metacercariae were obtained from Wuhu city of Anhui Province, China. The metacercariae were isolated through digestion of the fish with artificial gastric juice including pepsin (MP Biomedicals, Germany) and hydrochloric acid, and collected under a dissecting microscope. New Zealand White rabbits were infected intragastrically twice at an interval of 1 week and were sacrificed 6 weeks after the second infection. The livers were removed and *C. sinensis* adult worms were recovered from the bile duct. The adult worms were washed with phosphate-buffered saline (PBS) and kept in an −80 °C freezer until use.

### Structure prediction and expression of recombinant Cslegumain protein

Based on the previous bioinformatics analysis of Cslegumain, its secondary and tertiary structures were predicted using Phyre2 according to the amino acid sequences in this study. The fragment of Cslegumain was amplified according to the open reading frame using special forward and reverse primers containing the EcoRI or XhoI restriction enzyme site. Polymerase chain reaction (PCR) products were purified and inserted into the vector pET-28a(+) after digestion by the restriction enzymes EcoRI and XhoI. After sequencing, the recombinant plasmid was transformed into *Escherichia coli* BL21 (DE3) pLysS (Invitrogen, Carlsbad, CA, USA). After being induced by isopropyl β-d-thiogalactopyranoside (IPTG) at a final concentration of 1 mM at 37 °C for 4 h, *E. coli* cells were collected and lysed by sonication. The recombinant fusion protein was purified by Ni–nitrilotriacetic acid (NTA) affinity chromatography under denaturing conditions and eluted with imidazole and dialyzed against PBS. The purified protein was subjected to sodium dodecyl sulfate–polyacrylamide gel electrophoresis (SDS-PAGE), and the concentration was measured using the Bio-Rad protein assay (Bio-Rad, Philadelphia, PA, USA).

### Development of polyclonal antibody against Cslegumain and identification of native Cslegumain

BALB/c mice were immunized intraperitoneally with purified Cslegumain fusion protein (50 μg per mouse) emulsified with Freund’s complete adjuvant. Another two boosters of 50 μg recombinant protein emulsified with Freund’s Incomplete Adjuvant were performed at an interval of 14 days 2 weeks after the first immunization. The mice sera containing anti-Cslegumain polyclonal antibody was obtained 1 week after this third immunization. The soluble extract of *C. sinensis* adult worms was obtained as reported previously [13] and the recombinant Cslegumain protein was electrophoresed in 10% SDS-PAGE and transferred onto a nitrocellulose (NC) filter. After blocking with 5% skim milk, the membrane was incubated with mouse serum at a dilution of 1:1000 at 4 °C overnight and then with goat anti-mouse immunoglobulin G (IgG) alkaline phosphatase (AP)-conjugated antibody (Sigma, St. Louis, MO, USA) at room temperature for 2 h. The target protein was visualized by the substrate of BCIP/NBT (Sigma, St. Louis, MO, USA).

### Quantitative analysis of the developmental transcriptional level of Cslegumain

Total RNA was extracted from *C. sinensis* metacercariae and adult worms using TRIzol reagent (Ambion, Carlsbad, CA, USA) according to the manufacturer’s instructions. The first-strand complementary DNA (cDNA) was synthesized with oligo-d (T) primer using a Power cDNA Synthesis Kit (iNtRON Biotechnology, Gyeonggi-do, Korea) according to the manufacturer’s protocol. Quantitative real-time (qRT)-PCR was performed to detect the developmental transcriptional level of Cslegumain in metacercariae and adult worms. The Oligo 6 program was applied to design the primers, and the gene expressing phosphoglycerate kinase (PGK) was employed as a reference gene [14] (listed in Table 1). The reaction was performed using the LightCycler 1.5 (Roche, Mannheim, Germany) as follows: the reaction mixture was first heated to 95 °C for 15 min, followed by 45 cycles of 95 °C for 10 s, 60 °C for 10 s and 72 °C for 30 s. The relative transcriptional level was calculated using the 2^−△△Ct^ method.

**Table 1:** Primers sequences used to amplify the cDNAs by qRT-PCR

Primer	Sequence (5′ → 3′)	Size (base pairs)
Cslegumain F	CTTGCCTTCTCATTGCGTTCT	155
Cslegumain R	ATCTGCTTGGTGTCGGTAGTT	
Phosphoglycerate kinase F	GCGGGTGCTTA TGCGAGTTGA	190
Phosphoglycerate kinase R	CACCGGGTTGAGGGAA TA TCT	
E-cadherin F	GCTCTTCCAGGAACCTCTGTGATG	82
E-cadherin R	AAGCGATGGCGGCATTGTAGG	
N-cadheirn F	AAGGTGGATGAAGATGGCATGGTG	171
N-cadheirn R	TGCTGACTCCTTCACTGACTCCTC	
α-actinin 4 F	CCACCATTGCCCGCACCATC	133
α-actinin 4 R	ATGCTGCCTGTCTGCTTCTTGTC	
β-catenin F	GGCTCTTGTGCGTACTGTCCTTC	99
β-catenin R	GCTTCTTGGTGTCGGCTGGTC	
iNOS F	CAGGGTGGAAGCGGTAACAAAGG	86
iNOS R	CCTGCTTGGTGGCGAAGATGAG	
PI3K F	GCACGCCAAGGAATGCTACTAGG	168
PI3K R	GGACAGTAAGAACAGCCACCAACC	
AKT F	GCAGGATGTGGACCAACGTGAG	110
AKT R	GCAGGCAGCGGATGATGAAGG	

### Immunohistochemical staining of Cslegumain in *C. sinensis* adults

Rabbit liver infected with *C. sinensis* was fixed in 10% paraformaldehyde and embedded in paraffin and then sectioned into ribbons. After being deparaffinized and rehydrated, the ribbons were treated with anti-Cslegumain mouse sera or normal mouse sera at a dilution of 1:50 for 2 h at room temperature. After washing three times with PBS, the ribbons were incubated with polymer-horse radish peroxidase-labeled anti-mouse IgG antibody (Dako Cytomation, Glostrup, Denmark). Color was developed using 3-amino-9-ethylcarbazole as substrate, and the ribbons were counterstained with hematoxylin. Pictures were taken under a microscope.

### Cell culture, construction of the eukaryotic expression plasmid pcDNA3.1(+)-Cslegumain and cell transfection

The cholangiocarcinoma cell line, RBE cells (Procell, China), were cultured in RPMI 1640 (HyClone, USA) supplemented with 10% fetal bovine serum (FBS) (Gibco, USA) and 1% penicillin/streptomycin (Beyotime, China) with 5% CO_2_ at 37 °C. The Cslegumain fragment was amplified according to the open reading frame by PCR using the template of *C. sinensis* adult cDNA and specific forward and reverse primers containing *Hind*III and *EcoR*I restriction enzyme site. The PCR products were inserted into plasmid pcDNA3.1(+) (GenePharma, China) to construct pcDNA3.1(+)-Cslegumain, which was sequenced to ensure the correct insert. pcDNA3.1(+)-Cslegumain and pcDNA3.1(+) were transfected into RBE cells by Lipofectamine™ 2000 (Invitrogen, USA), respectively. The transfection efficiency was checked by a green fluorescent protein tag, and the expression of Cslegumain was analyzed by western blotting incubated with mouse anti-Cslegumain sera.

### Cell proliferation assay (CCK-8)

A Cell Counting Kit-8 (CCK-8, MedChemexpress, USA) assay was performed to assess cell proliferation and activity according to the manufacturer’s instructions. Briefly, each group of Cslegumain-expressing and pcDNA3.1(+) transfected RBE cells suspension was adjusted to 1 × 10^5^ cells/ml. Then, 100 μl cell suspension was cultured in each well of a 96-well plate for 4 h and CCK-8 solution was added. The cells were cultured for 0 h, 24 h, 48 h and 72 h, respectively. The absorbance of each well at 450 nm was recorded by microplate reader (Eppendorf, Germany) at the above four time points.

### Scratch wound healing assay

A total of 3 × 10^5^ RBE cells in each group of Cslegumain-expressing cells, pcDNA3.1(+)-transfected cells and control RBE cells were seeded into six-well plates and scraped with the fine end of a 10-μl pipette tip (time 0 h) when the cells grew to 90% confluence. Cell migration was photographed at 0 h, 24 h and 48 h, respectively, after injury. Wound healing was measured as area change of induced injury compared with the 0-h control.

### Matrigel transwell assay

The RBE cells were suspended with serum-free RPMI 1640 medium and added to the upper chamber of the transwell insert coated with matrix glue Matrigel (Corning, USA) containing 8-µm pores. The insert was put into the lower chamber in the 24-well plates with 20% FBS in RPMI 1640. After incubation for 48 h, the cells in the upper chamber were removed. The cells invading to the bottom surface were fixed with 4% formaldehyde and stained with tertiary violet. The invasion rate was calculated by counting all the invading cells in the bottom surface under ×10 magnification.

### RNA extraction and qRT-PCR

The cultured RBE cells were collected by centrifuge 48 h after transfection and the total RNA was extracted using TRIzol reagent (Sangon Biotech, Shanghai, China). The first-strand cDNA was synthesized using a Power cDNA Synthesis Kit (SparkJade, China) according to the manufacturer’s instructions. Quantitative real-time PCR was performed to detect the developmental transcriptional level of E-cadherin, N-cadherin, PI3K, AKT, β-catenin, α-actinin 4 and inducible nitric oxide synthase (iNOS). The glyceraldehyde-3-phosphate dehydrogenase (GAPDH) was employed as a reference gene and all the primers were designed by Sangon Biotech, Shanghai, China (listed in Table 1). The reaction was performed using CFX96 (Bio-Rad, USA) as follows: the reaction mixture was first heated to 95 °C for 30 s, followed by 41 cycles of 95 °C for 15 s, 55 °C for 30 s and 72 °C for 20 s. The transcriptional level was calculated using the 2^−△△Ct^ method.

### Western blotting

Total protein was extracted from the above RBE cells using a protein extraction kit (SparkJade, China) according to the manufacturer's instructions and the protein concentration was measured using Bio-Rad Protein Assay (Bio-Rad, Philadelphia, PA, USA). The proteins were separated by 10% SDS-PAGE and transferred onto a nitrocellulose filter. The membrane was blocked within 5% skim milk for 1 h at room temperature and then incubated with rabbit anti-human E-cadherin, N-cadherin, β-catenin, α-actinin 4 and GAPDH monoclonal antibodies (Abcam, USA) at 4 °C overnight. After washing, the membrane was incubated with horseradish peroxidase-conjugated goat anti-rabbit IgG antibody for 2 h at room temperature. Protein bands were visualized by enhanced chemiluminescence (ECL) luminescent liquid (SparkJade, China).

### Statistical analysis

All data were analyzed using SPSS 24.0 and were expressed as the mean ± standard error of the mean (SEM). The *t*-test was used to analyze the significance of differences between groups in cell proliferation and qRT-PCR. One-way analysis of variance (ANOVA) was employed to analyze the significance of differences among groups in scratch wound healing assay and Matrigel transwell assay. All results were considered significantly significant when *P* < 0.05.

## Results

### Secondary and tertiary structures of Cslegumain and identification of recombinant and native Cslegumain

**Fig. 1:**
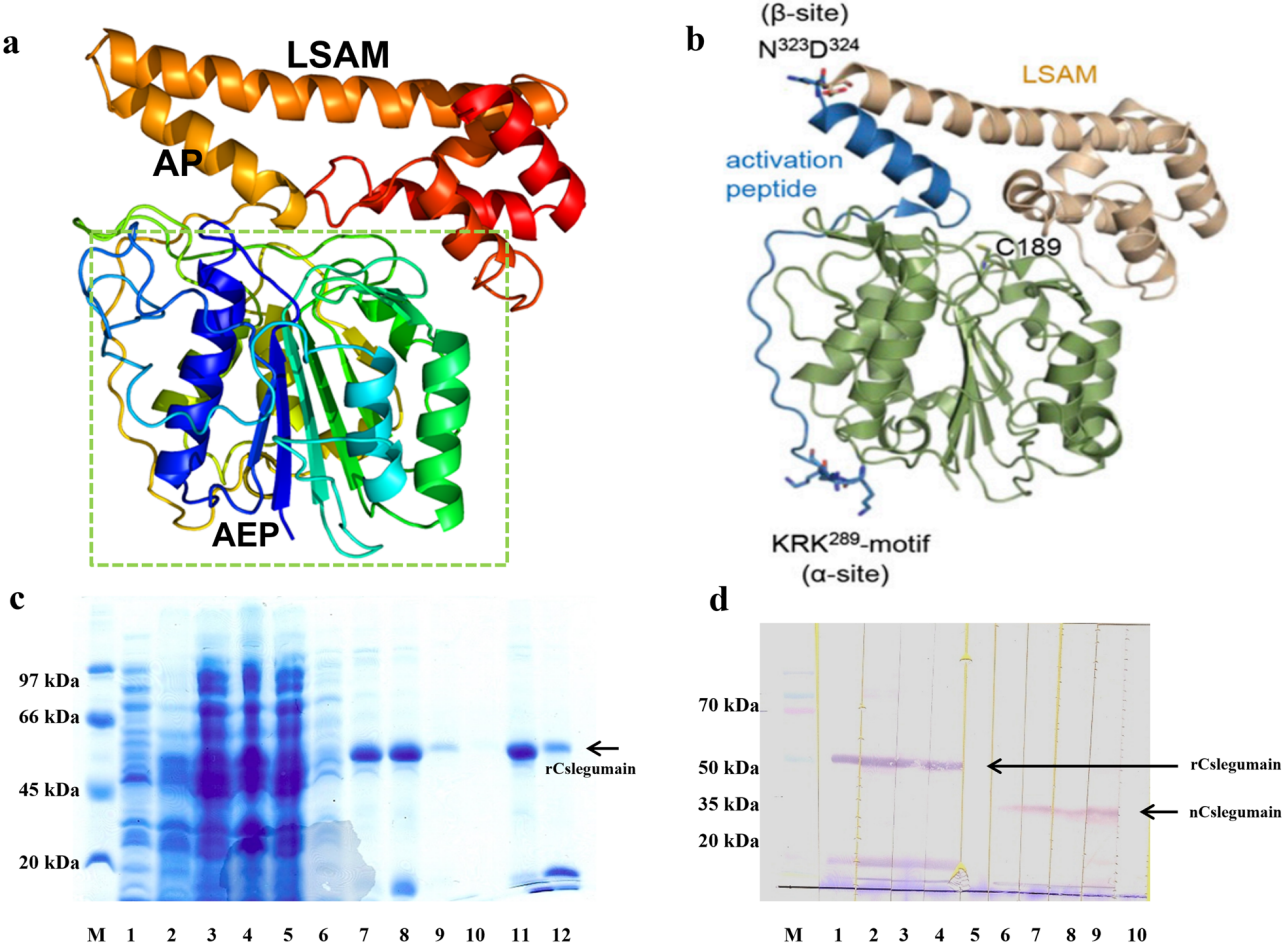
The basic information of Cslegumain. **a** Predicted tertiary structure of Cslegumain. Cslegumain showed a very similar tertiary structure as Hlegumain, with a catalytic AEP domain (in the green frame) and a C-terminal prodomain composed of AP domain and LSAM domain. **b** The tertiary structure of Hlegumain (Elfriede et al., 2013). **c** Purified recombinant Cslegumain from *E. coli*. The arrow shows the purified protein band. M, marker; lane 1, uninduced; lane 2, induced total; lane 3, supernatant; lane 4, pellet; lane 5, pass through; lane 6, washing; lanes 7–12, eluted and purified Cslegumain. (**d**) Detection of recombinant and native Cslegumain. Lanes 1–4, immunized mouse sera recognized recombinant Cslegumain; lane 5, normal mouse sera did not react with the recombinant protein. Lanes 6–9, immunized mouse sera recognized native Cslegumain; lane 10, normal mouse sera did not react with native Cslegumain

Multiple alignment of a deduced amino acid sequence of proCslegumain with other organisms was reported previously [12]. However, its secondary and tertiary structures were not reported. Therefore, in the present study, the secondary and tertiary structures of proCslegumain were predicted using the online program Phyre2. Through alignment, the proCslegumain showed a very similar secondary structure as human legumain from K35, with helixes and sheets in almost the same position (Additional file 1: Fig. S1) [15]. For the tertiary structure, proCslegumain also consisted of a catalytic AEP domain and a C-terminal prodomain, which can be further segmented into a latency-conferring activation domain (AP) and an LSAM (legumain stabilization and activity modulation domain) (Fig. 1a and 1b). For representative Protein Data Base (PDB) templates see c4fguB, c4awaA, c5nijB and c6ysaH.

To obtain the anti-Cslegumain mouse sera, firstly, the fusion protein of Cslegumain with a 6 × his tag was induced to express in *E. coli* BL21 and purified by Ni-NTA agarose efficiently with high yield and purity. This purified recombinant Cslegumain protein was used for immune sera production from mice. Western blot was performed to identify the recombinant and native Cslegumain. The mouse sera recognized recombinant Cslegumain with approximate molecular masses of 50 kDa and native Cslegumain from *C. sinensis* soluble extract with only one band of estimated molecular mass of 36 kDa (Fig. 1c and d). These results indicated that Cslegumain shared a similar structure and domains with Hlegumain and is expressed in an active form in *C. sinensis* adults.

### Developmental transcriptional level of Cslegumain

**Fig. 2:**
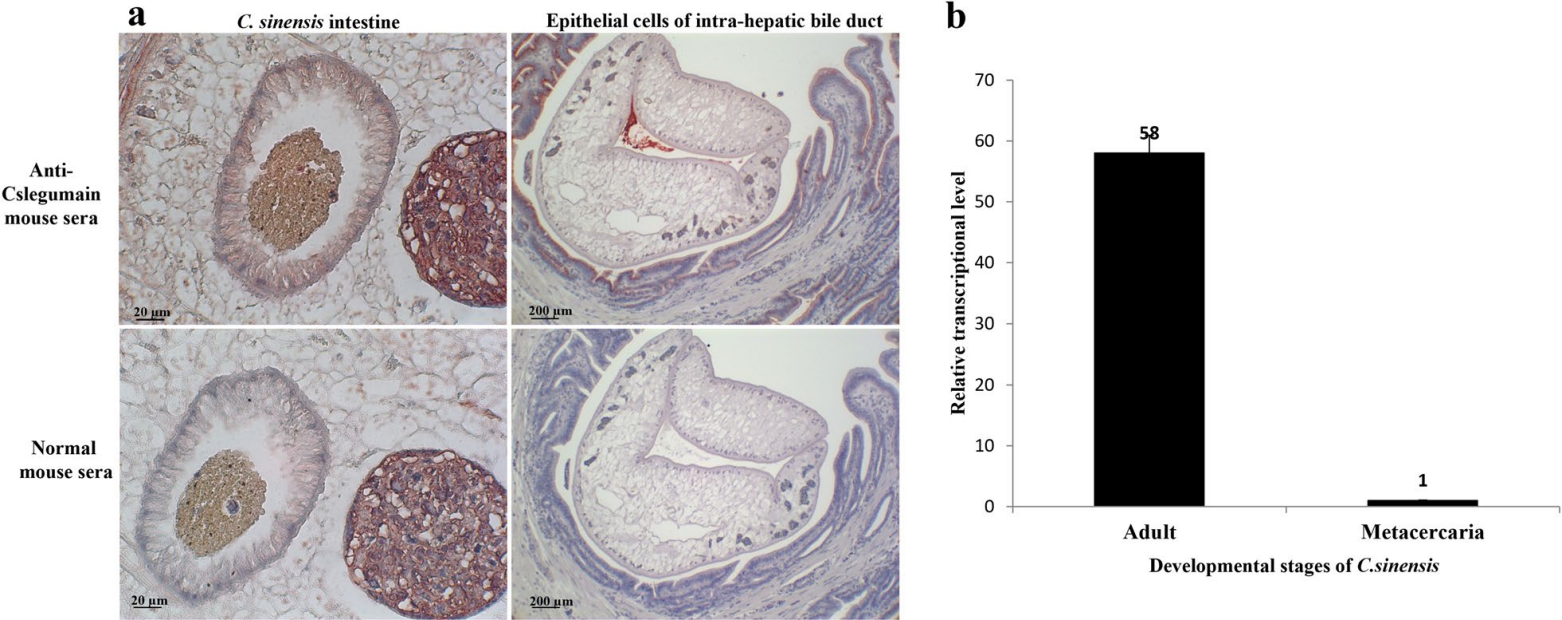
Localization of Cslegumain in *C. sinensis* and its relative transcriptional level in different developmental stages. **a** Localization of Cslegumain in *C. sinensis* adults. Positive signals were detected in the intestine and epithelial cells of intrahepatic bile ducts. **b** Relative transcriptional levels of Cslegumain in *C. sinensis* adults and metacercariae analyzed by qRT-PCR, the Cslegumain showed higher transcriptional levels in adults than in metacercariae of *C. sinensis*

ESP components have diverse functions due to their expression in different stages. In order to elucidate the main worm stage in which Cslegumain plays a role, qRT-PCR was performed using total RNA as a template to analyze the transcriptional level of Cslegumain in the developmental stages of *C. sinensis* metacercariae and adults. The relative transcriptional level of Cslegumain was calculated according to cycle threshold (C_T_) values of Cslegumain and the reference gene of PGK using the 2^−△△Ct^ method. The result showed that the Cslegumain level was 58 times as high in the adults as in the metacercariae (Fig. 2b). This finding indicated that Cslegumain was expressed mainly in the adult stage of *C. sinensis* and also caused pathological damage to the host in this stage.

### Distribution of Cslegumain in *C. sinensis* adult

Immunohistochemical staining using mouse sera revealed that Cslegumain was localized in the intestine. However, positive signals were also observed in the epithelial cells of the intrahepatic bile duct, indicating that Cslegumain was secreted into the surroundings and might play various roles in causing pathological changes in the bile duct (Fig. 2a).

### Influence of Cslegumain on proliferation and activity of cholangiocarcinoma RBE cells

**Fig. 3:**
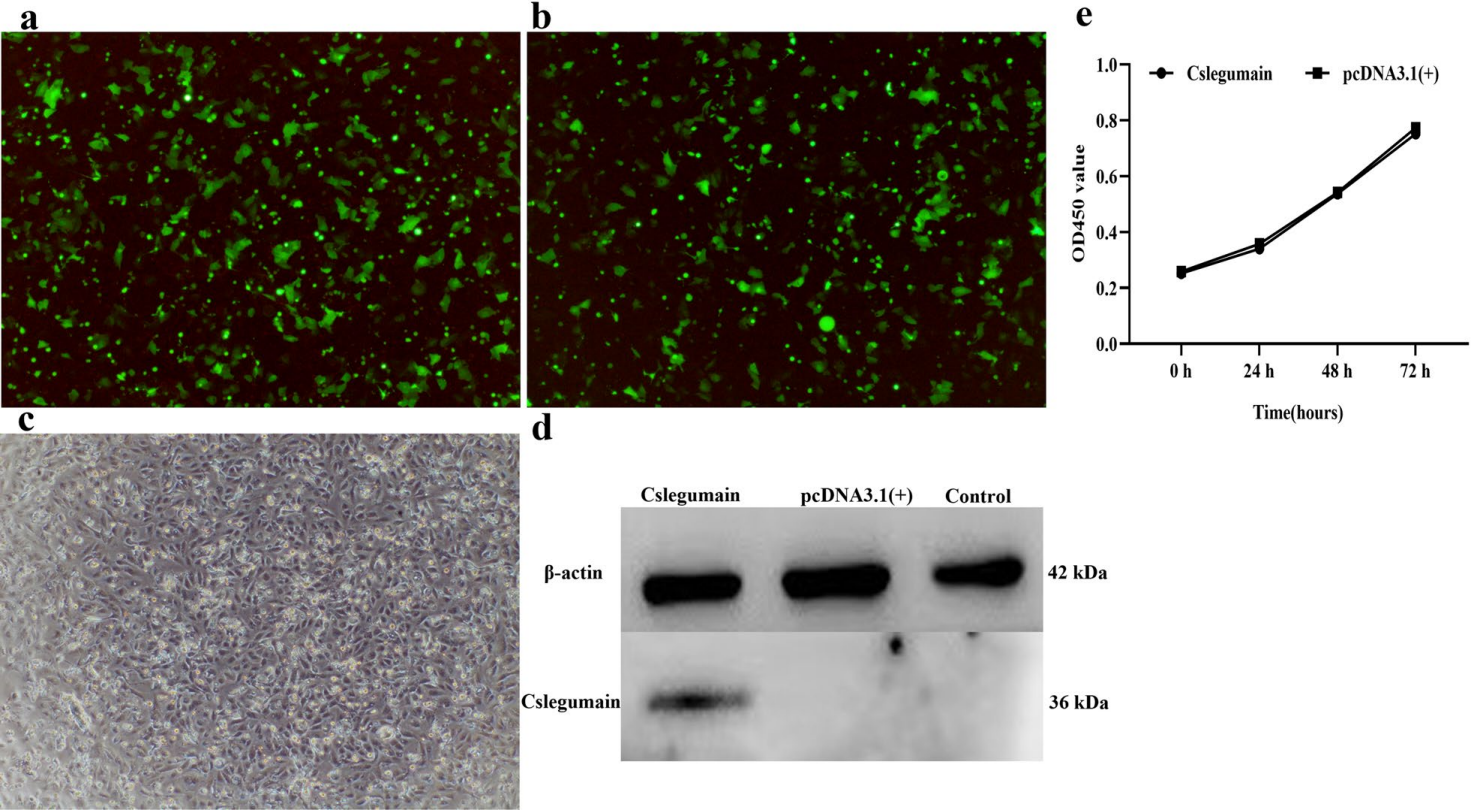
Transfection and expression of Cslegumain in RBE cells and effects on the proliferation of RBE cells. **a** pcDNA3.1-Cslegumain. **b** pcDNA3.1(+). **c** Control RBE cells. **d** Western blot assay of Cslegumain. **e** Effects of Cslegumain on the proliferation of RBE cells. The CCK-8 assay showed that Cslegumain had no significant influence on the proliferation of RBE cells from 24 to 72 h after culture (*P* > 0.05)

Since *C. sinensis* ESP play an important role in promoting cholangiocarcinoma, as a member of ESP, whether Cslegumain accelerates the proliferation of tumor cells should be clarified. In order to investigate the influence of Cslegumain on cholangiocarcinoma cells, the cDNA fragment of Cslegumain was inserted into plasmid pcDNA3.1(+) and transfected into RBE cells. The transfection efficiency was high, about 90% (Fig. 3a–c). The anti-Cslegumain mouse sera recognized the expressed Cslegumain in RBE cells, while no band appeared in the control or pcDNA3.1(+)-transected RBE cells (Fig. 3d). CCK-8 assay was performed to assess the proliferation and activity of RBE cells. The results showed that there was no significant difference in the proliferation and activity between pcDNA3.1(+)-Cslegumain, pcDNA3.1(+)-transfected cells and control RBE cells (*P* > 0.05) (0 h: *t*-test, *t*_(4)_ = −0.135, *P* = 0.899; 24 h: *t*-test, *t*_(4)_ = −0.399, *P* = 0.710; 48 h: *t*-test,* t*_(4)_ = −0.053, *P* = 0.960; 72 h: *t*-test, *t*_(4)_ = −0.255, *P* = 0.811) (Fig. 3e). Thus, we concluded that Cslegumain would not promote RBE cell growth.

### Cslegumain promotes the invasion and migration of cholangiocarcinoma RBE cells

**Fig. 4:**
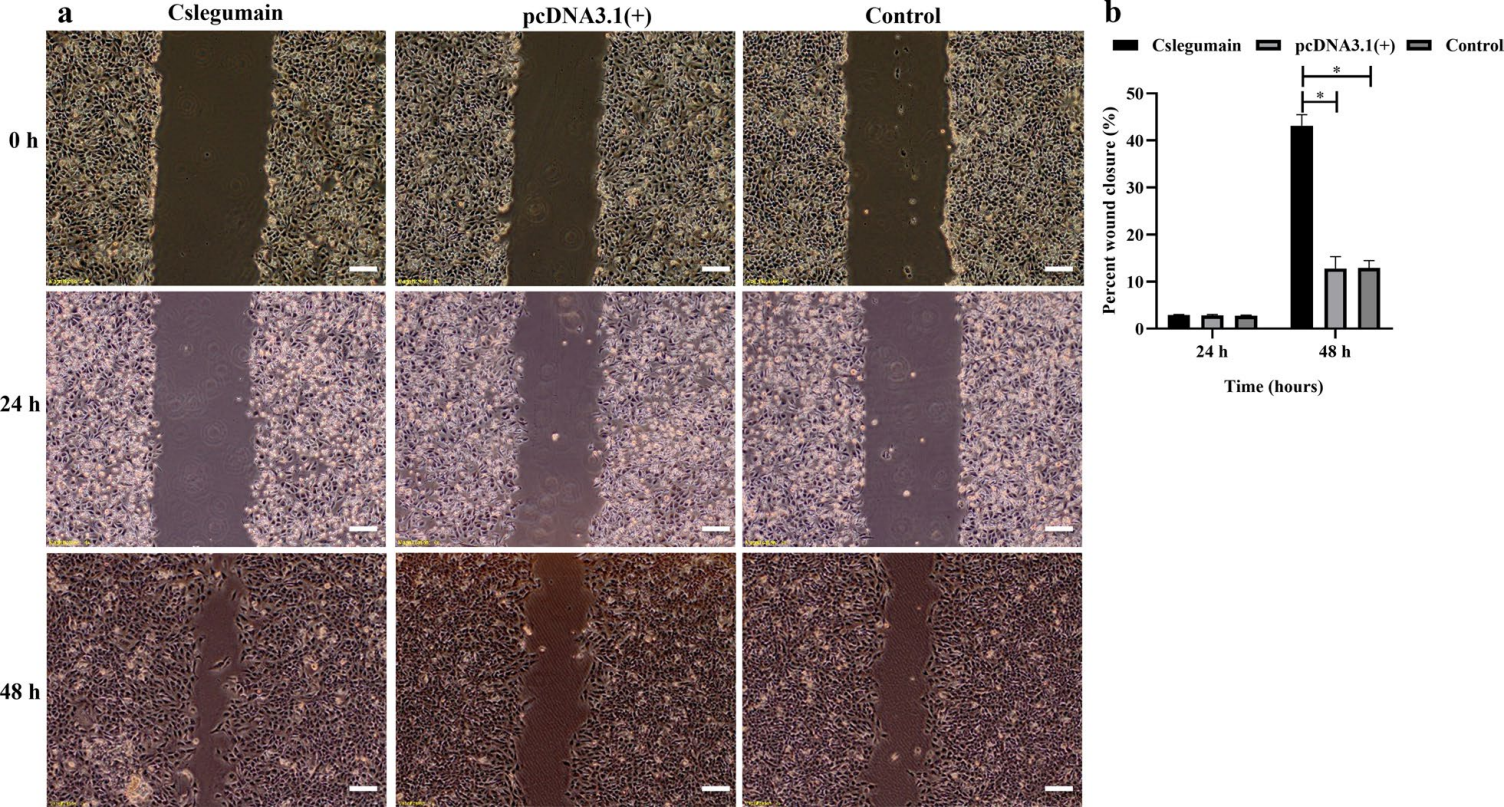
Effects of Cslegumain on the migration ability of RBE cells. **a** Wound healing assay of Cslegumain expression, pcDNA3.1(+)-transfected and control RBE cells at 0 h, 24 h and 48 h after culture (scale bar 200 μm). **b** The wound closure percentages. The figure shows that the Cslegumain-expressing RBE cells displayed faster wound healing than pcDNA3.1(+)-transfected and control RBE cells at 48 h but not at 24 h after injury (*P < 0.05)

**Fig. 5:**
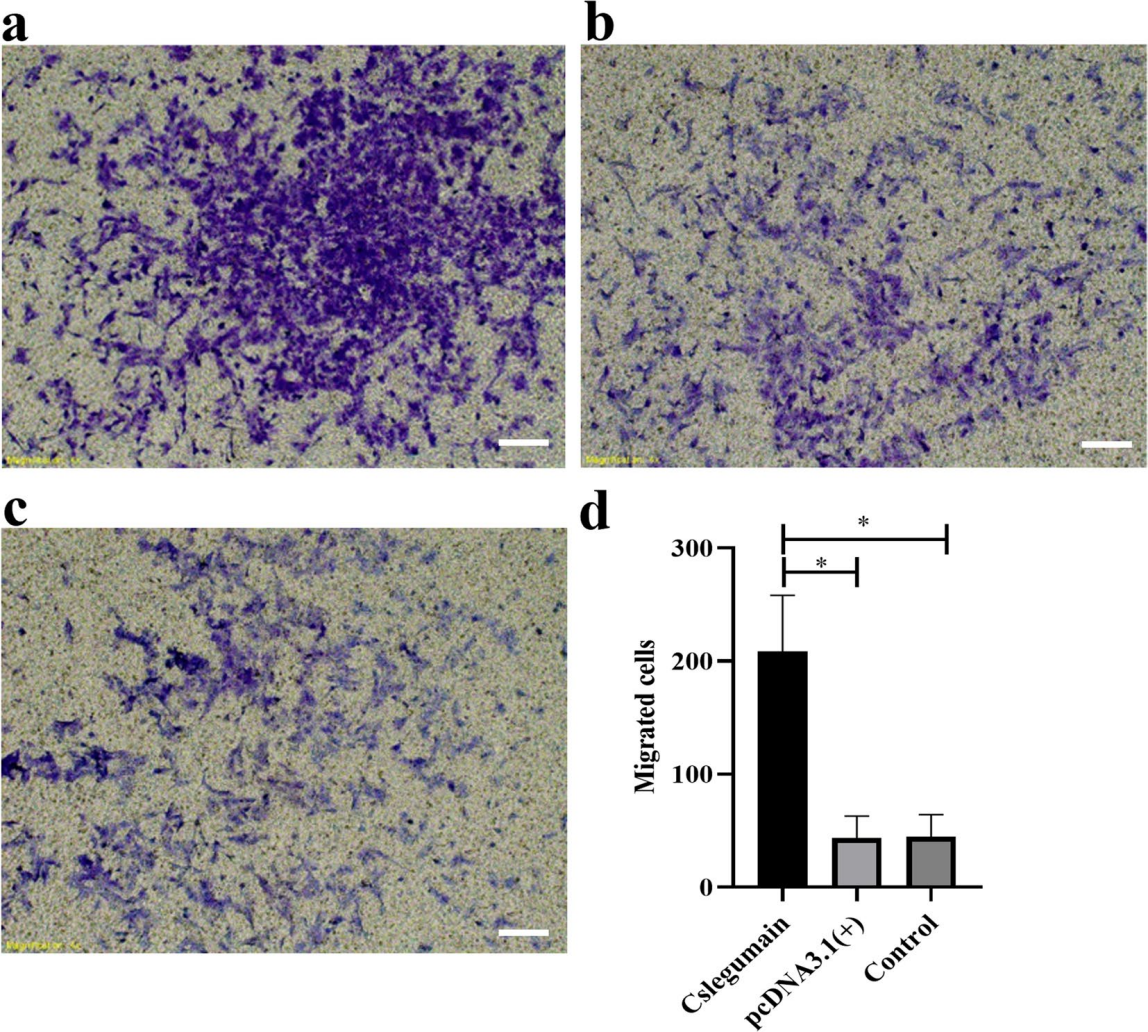
Effects of Cslegumain on the invasion ability of RBE cells. **a** Cslegumain-expressing cells that invaded to the bottom surface (scale bar: 200 µm). **b** pcDNA3.1(+)-transfected cells that invaded to the bottom surface. **c** Control RBE cells that invaded to the bottom surface. **d** The number of cells was counted under ×10 magnification. Values were compared using one-way ANOVA. The finding showed that the number of Cslegumain-expressing cells was significantly higher than that in pcDNA3.1(+)-transfected and control RBE cells after 36 h (*P < 0.05)

Besides proliferation, invasion and migration were very important mechanisms for tumor development. To further evaluate whether Cslegumain promotes cholangiocarcinoma cell migration, a wound healing assay was carried out. Compared with pcDNA3.1(+)-transfected cells and control RBE cells, Cslegumain-overexpressing RBE cells displayed faster wound healing at 48 h (ANOVA, *F*_(2, 6)_ = 195.165, *P* < 0.0001) but not at 24 h after injury (*P* > 0.05) (ANOVA, *F*_(2, 6)_ = 1.944, *P* = 0.223) (Fig. 4). For cell invasion, a Matrigel transwell assay was performed. The result indicated that Cslegumain increased the invasive ability of RBE cells significantly (ANOVA,* F*_(2, 6)_ = 25.002, *P* = 0.006) (Fig. 5).

### Cslegumain induced upregulation of EMT molecules in RBE cells

**Fig. 6:**
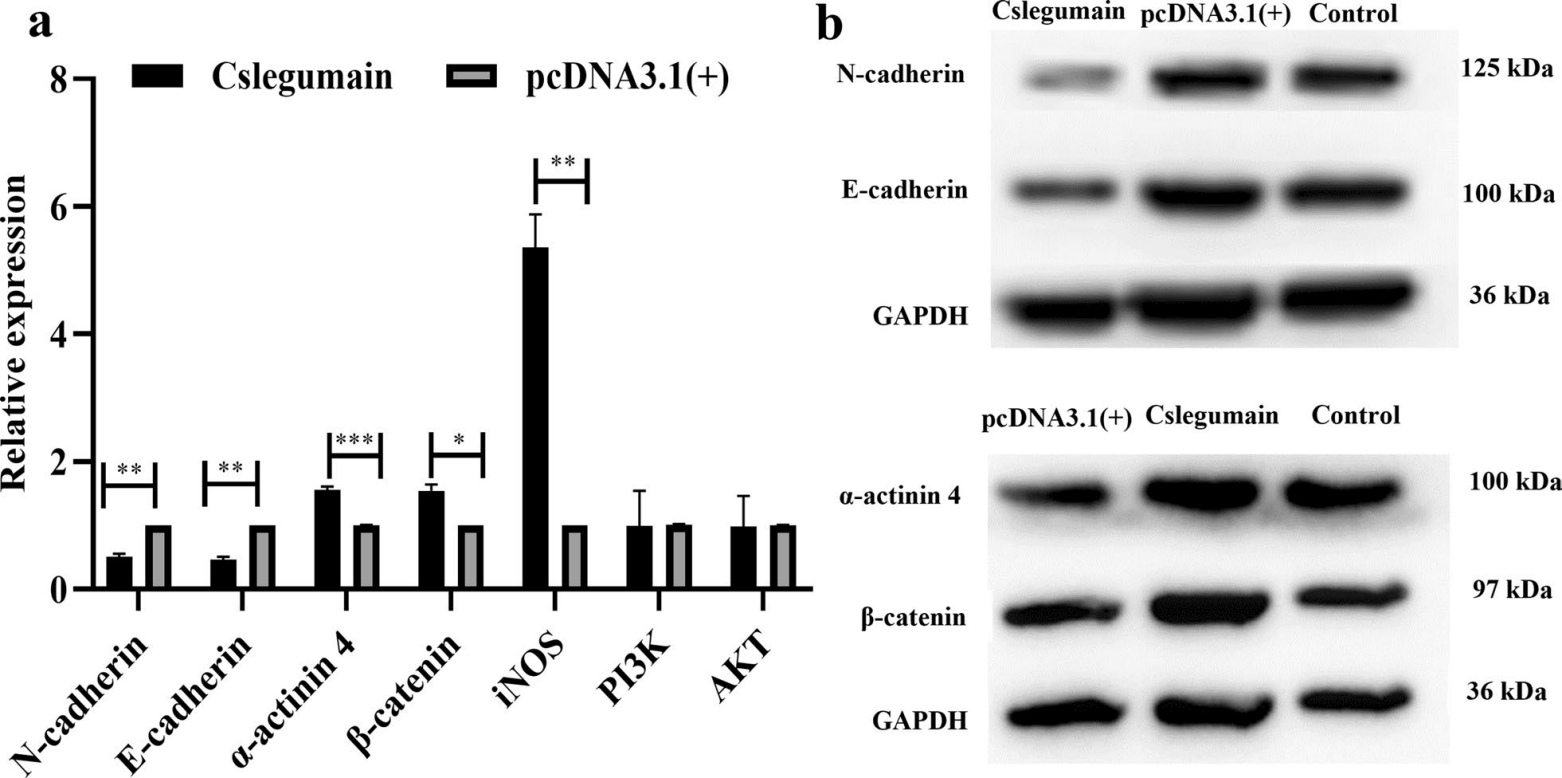
Effects of Cslegumain on expression levels of tumor-related molecules. **a** Relative transcriptional levels of the tumor-related molecules analyzed by qRT-PCR. Values were compared using one-way ANOVA. Compared with pcDNA3.1(+)-transfected and control RBE cells, Cslegumain-expressing cells displayed decreased transcriptional levels of E-cadherin and N-cadherin as well as increased α-actinin4, β-catenin and iNOS 48 h after culture. However, the levels of PI3K and AKT showed no significance among the three groups (*P < 0.05; **P < 0.01; ***P < 0.001). **b** Effects of Cslegumain on protein levels of E-cadherin, N-cadherin, actinin4 and β-catenin. The results indicated that Cslegumain downregulated the protein levels of E-cadherin and N-cadherin and upregulated the level of actinin 4 and β-catenin

Based on the above fact that Cslegumain promoted cholangiocarcinoma RBE cell migration and invasion, the molecular mechanisms were further investigated in our study. qRT-PCR was performed to assess the transcriptional levels of molecules including E-cadherin, N-cadherin, α-actinin 4, β-catenin and iNOS which play an important role in epithelial–mesenchymal transition (EMT). Compared with the pcDNA3.1(+)-transfected and control RBE cells, the Cslegumain-expressing cells displayed decreased transcriptional levels of E-cadherin and N-cadherin along with increased transcriptional levels of α-actinin 4, β-catenin and iNOS 48 h after transfection (E-cadherin: *t*-test, *t*_(4)_ = −21.174, *P* = 0.002; N-cadherin: *t*-test, *t*_(4)_ = −17.603, *P* = 0.003; α-actinin 4: *t*-test, *t*_(4)_ = 16.899, *P* < 0.0001; β-catenin: *t*-test, *t*_(4)_ = 9.151, *P* = 0.012; iNOS: *t*-test,* t*_(4)_ = 14.326, *P* = 0.005) (Fig. 6a). However, the levels of PI3K and AKT displayed no significance among the three groups (PI3K: *t*-test, *t*_(4)_ = −0.064, *P* = 0.952; AKT: *t*-test,* t*_(4)_ = 0.142, *P* = 0.941) (Fig. 6a). Besides the mitochondrial RNA (mRNA), the protein levels of E-cadherin, N-cadherin, α-actinin 4 and β-catenin were detected by western blotting and showed the same trends with mRNA changes. Collectively these findings demonstrated that Cslegumain could increase the invasion and migration ability of RBE cells through regulating the expression levels of E-cadherin, N-cadherin, α-actinin 4, β-catenin and iNOS (Fig. 6b).

## Discussion

The ESP of *C. sinensis* were proved to play important roles in the oncogenesis and development of cholangiocarcinoma [16]. Pak et al. found that the *C. sinensis* ESP could change the expression levels of various proteins in cholangiocarcinoma cells, leading to the occurrence and development of cholangiocarcinoma [17]. Pak et al. found that the *C. sinensis* ESP could change the microRNA (miRNA) levels in human cholangiocarcinoma cells which were associated with tumor cell proliferation, migration and invasion through the regulation of oncogenes [18]. Because different components of ESP may promote the progression of cholangiocarcinoma via various mechanisms, studies to identify more functional ESP components are warranted. In the present study, we analyzed the secondary and tertiary structures of Cslegumain and found that they showed high similarity with the structures of Hlegumain. After western blotting analysis, only the catalytic 36 kDa N-terminal mature enzyme was detected in the *C. sinensis* total proteins, which was different from the three bands of 22 kDa, 36 kDa and 58 kDa found in an earlier study [12]. With the change in *C. sinensis* developmental stages, different protein expression profiles were presented, which may play diverse roles in causing pathological changes. Cslegumain showed higher transcriptional levels in the adult worms than in the metacercariae, which was consistent with Jung’s study and indicated that Cslegumain might produce an effect mainly in the adult worm.

Proteins with similar amino acid sequences and structures may have the same or similar biological functions. Hlegumain was proved to be closely related to the oncogenesis or progression of various solid tumors [11]. Hlegumain promoted cervical cancer invasion and metastasis through the degradation of extracellular matrix by activating MMP-2 and induced endothelial permeability and tumor metastasis by downregulating ZO-1 [19, 20]. Thus, Hlegumain plays important roles in tumor metastasis and invasion. In a previous study, we found that Hlegumain obviously promoted the migration and invasion of cholangiocarcinoma cells (which was not published). Since Cslegumain showed very similar secondary and tertiary structures as Hlegumain, whether it also plays equivalent roles in cholangiocarcinoma deserves further investigation. In the present study, we inserted the open reading frame into plasmid and transfected cholangiocarcinoma RBE cells. The results showed that Cslegumain obviously promoted the migration and invasion ability of the cells, indicating the similar functions of Cslegumain and Hlegumain in promoting the progression of cholangiocarcinoma. Hlegumain was also found to promote the proliferation and invasiveness of prostate cancer cells via the PI3K/AKT signaling pathway [21]. However, in our study, Cslegumain was not found to be related to RBE cell proliferation, with no mRNA level significance of PI3K/AKT demonstrating the diverse function of Hlegumain.

EMT plays critical roles in oncogenesis and development of tumors by promoting the invasive and metastatic activity of malignant tumor cells. A variety of molecules including E-cadherin, N-cadherin and Snail are involved in EMT. E-cadherin is a protein present in the membrane of an epithelial cell and forms the "zipper" structure to "stick" cells together. E-cadherin was found to be downregulated or missing in the metastasis of many tumor cells including breast cancer, gastric cancer and cervical cancer [22, 23]. The loss of E-cadherin is regarded as a marker of EMT and promotes metastasis not only by breaking the junction between cells but also by inducing multiple downstream transcriptional pathways [24]. In our study, the expression of E-cadherin was also decreased in Cslegumain-transfected RBE cells, indicating that Cslegumain might increase RBE cell motility through downregulation of E-cadherin, which showed the same regulation as legumain to E-cadherin in gastric cancer [25].

N-cadherin, another cell adhesion molecule, is mainly distributed in nerve tissue, striated muscle and myocardial tissue and is involved in regulating calcium-mediated adhesion between cells of the same type, cell aggregation and migration. In some studies, N-cadherin was considered to be positively correlated with tumor invasion and metastasis, such as in breast cancer, cervical carcinoma and prostate cancer [26–29]. However, in other studies, N-cadherin was found to show low expression in osteosarcoma, glioma, serous ovarian cancer and liver cancer [30–32]. The loss of N-cadherin was highly related to the recurrence of liver cancer [33]. The heterogeneity of N-cadherin expression may depend on various tumors or tissues [34]. In our study, the expression of N-cadherin was downregulated, indicating that Cslegumain plays the same roles in promoting cell migration and invasion ability via downregulation of N-cadherin (human legumain also downregulated N-cadherin in our study, which was not reported). Our study displayed the opposite function of N-cadherin in tumor progression as the study by Sutthiwan [35], which may be due to different tumor types and deserves further investigation.

The canonical Wnt-β-catenin signaling pathway plays important roles in many biological processes including regulation of cell proliferation, differentiation and apoptosis, and was also found to be closely related to oncogenesis and tumor progression [36]. For cholangiocarcinoma, the Wnt-β-catenin signaling pathway often interacts with certain genes to increase their pro-tumorigenic effect in growth and migration and finally enhances the refractory characters. Inhibition of Wnt-β-catenin signaling induces cell apoptosis and suppresses cell proliferation in cholangiocarcinoma cells [37]. When the Wnt-β-catenin signaling pathway is activated, as a central effector, β-catenin translocates into the nucleus and regulates target genes to control cell proliferation, differentiation and even EMT [38]. Legumain (AEP) was reported to promote the invasion and metastasis of gastric cancer cells by modulating EMT and promoting β-catenin expression [25]. In our study, Cslegumain also promoted the invasion and migration of cholangiocarcinoma cells by increasing β-catenin levels, indicating the same function as Hlegumain.

α-Actinin 4 (ACTN4), a member of the spectrin superfamily, plays an important role in maintaining cytoskeletal integrity and controlling cell motility. High expression of α-actinin 4 was demonstrated to be involved in cell adhesion and invasion through cytoskeletal reorganization [39, 40]. Studies also showed that α-actinin 4 could promote EMT and was closely related to low expression of E-cadherin and upregulation of β-catenin in tumor invasion and metastasis [40]. In cholangiocarcinoma, α-actinin 4 was also overexpressed in tumor tissues and cell lines and was considered to be a candidate marker [41]. In our study, α-actinin 4 was also upregulated by Cslegumain, indicating that Cslegumain could promote cholangiocarcinoma cell migration through regulation of α-actinin 4. The mechanism by which Cslegumain regulates α-actinin 4 and the interaction between them need to be clarified in the future.

Inflammatory microenvironments play a critical role in DNA damage, carcinogenesis and tumor invasion. The pro-inflammatory enzyme, inducible nitric oxide synthase (iNOS), is responsible for production of NO and has been found to be correlated with oncogenesis, metastasis and malignancy in a variety of tumors such as gastric cancer, hepatocellular carcinoma and melanoma [42–45]. iNOS could also lead to the downregulation of E-cadherin in EMT leading to the decrease of intercellular adhesion and tumor metastasis [46]. In intrahepatic cholangiocarcinoma, iNOS was identified to be upregulated and was closely related to vessel invasion and lymph node metastasis [47]. In the present study, the transcriptional level of iNOS was upregulated by Cslegumain in RBE cells suggesting that Cslegumain might also promote the metastasis and invasion of cholangiocarcinoma cells through the regulation of iNOS.

## Conclusions

*Clonorchis sinensis* infection causes injury to the bile duct and liver and eventually leads to cholangiocarcinoma. *Clonorchis sinensis* ESP play critical roles in the oncogenesis and progression of cholangiocarcinoma. However, the components and precise mechanism remain unclear and deserve further investigation. Here, the Cslegumain displayed very similar secondary and tertiary structures as human legumain, indicating their same or similar function in tumors. As it is secreted into the surroundings, Cslegumain may play various roles in cholangiocarcinoma. In this study, it was demonstrated that Cslegumain could promote the invasion and migration of cholangiocarcinoma cells through downregulation of E-cadherin and N-cadherin and upregulation of α-actinin 4, β-catenin and iNOS. This study provides new insight into the mechanism of cholangiocarcinoma caused by *C. sinensis*.

## Supplementary Information

**Additional file 1: Figure S1.** Predicted secondary structure of proCslegumain. The predicted secondary structure represents proCslegumain. ProCslegumain shows a very similar structure as the templates, with almost the same helixes or sheets in the same positions. G = 3-turn helix (310 helix). I = 5-turn helix (π helix). T = hydrogen-bonded turn. B = residue in isolated β-bridge. S = bend. Identical residues in the alignment are highlighted with a gray background.

## Data Availability

Data supporting the conclusions of this article are included within the article.

## Abbreviations

*C. sinensis*: *Clonorchis sinensis*
ESP: Excretory–secretory products
Cslegumain: *C. sinensis* legumain
Hlegumain: Human legumain
MMP-2: Matrix metalloproteinase 2
MMP-9: Matrix metalloproteinase 9
SDS-PAGE: Sodium dodecyl sulfate polyacrylamide gel electrophoresis
PGK: Phosphoglycerate kinase
FBS: Fetal bovine serum
cDNA: Complementary deoxyribonucleic acid
CCK-8: Cell counting kit-8
qRT-PCR: Quantitative real-time polymerase chain reaction
iNOS: Inducible nitric oxide synthase
GAPDH: Glyceraldehyde-3-phosphate dehydrogenase
ACTN4: α-Actinin 4
EMT: Epithelial–mesenchymal transition
ECL: Enhanced chemiluminescence
