# Supplementary figures and images for: Clonorchis sinensis legumain promotes migration and invasion of cholangiocarcinoma cells via regulating tumor-related molecules
Source: Parasit Vectors. 2023 Feb 16;16:71. doi: 10.1186/s13071-023-05694-4 (PMC9933405; doi:10.1186/s13071-023-05694-4)

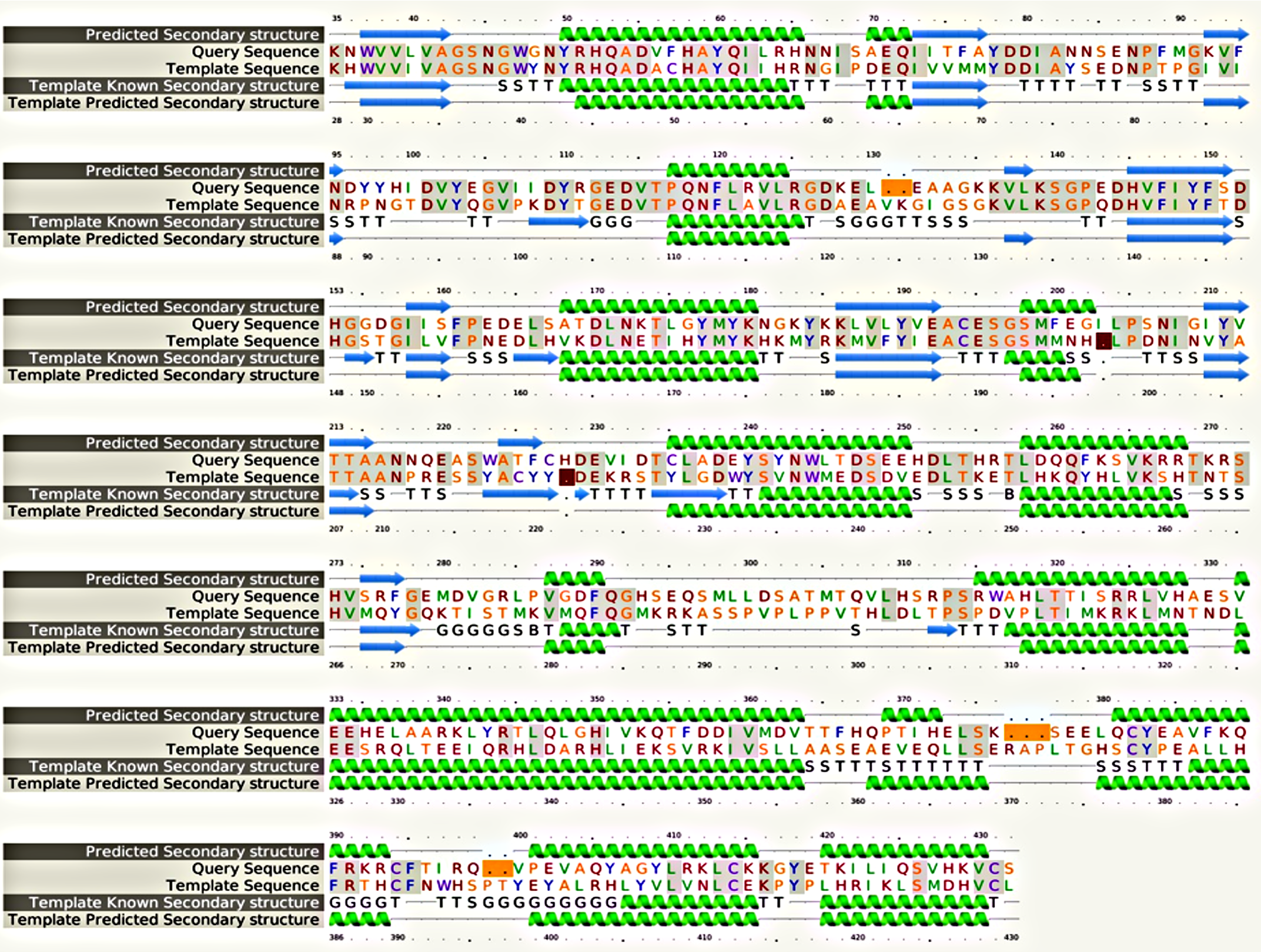

Supplement: Supplementary file 1 — Additional file 1: Figure S1. Predicted secondary structure of proCslegumain. The predicted secondary structure represents proCslegumain. ProCslegumain shows a very similar structure as the templates, with almost the same helixes or sheets in the same positions. G = 3-turn helix (310 helix). I = 5-turn helix (π helix). T = hydrogen-bonded turn. B = residue in isolated β-bridge. S = bend. Identical residues in the alignment are highlighted with a gray background. [file 13071_2023_5694_MOESM1_ESM.tif]
